# Facilitators and barriers to smoking cessation: a qualitative study among health professionals in Germany

**DOI:** 10.1186/s12913-025-12646-4

**Published:** 2025-04-01

**Authors:** Frederike Bokemeyer, Paulina Kiefer, Lea Schmidt, Kathleen Gali

**Affiliations:** 1 Department of Medical Psychology, University Medical Center Hamburg, Hamburg, Germany; 2 Center for Oncology, II. Medical Clinic and Polyclinic, University Medical Center Hamburg Eppendorf, Hamburg, Germany; 3 Cancer Epidemiology and Prevention Group, University Cancer Center Hamburg (UCC Hamburg), University Medical Center Hamburg-Eppendorf (UKE), Hamburg, Germany; 4 Hamburg Center for Health Economics (HCHE), University of Hamburg, Hamburg, Germany

**Keywords:** Smoking cessation, Tobacco, Smoking, Health professionals, Qualitative

## Abstract

**Background:**

Tobacco consumption remains a leading cause of global morbidity and mortality and is a significant preventable health concern. Despite the known benefits of smoking cessation, many smokers face difficulties in maintaining abstinence and preventing relapse. In Germany, approximately 30% of individuals aged 14 and older are smokers, which reflects low smoking cessation rates and limited use of evidence-based smoking cessation interventions.

**Purpose:**

This qualitative study aimed to explore experts’ views on smoking cessation through interviews with health practitioners.

**Methods:**

Fifteen semi-structured in-depth interviews were conducted with professionals from diverse fields, including medical doctors, psychologists, and addiction therapists, from July to November 2022. The data were analyzed using qualitative content analysis. A deductively developed categorization system was applied to identify sub-themes within categories and to systematically code the data. All data were thencategorized under two main categories: facilitators and barriers to smoking cessation.

**Results:**

Key facilitators included the self-motivation of participants, the communication skills of the intervention leader, and the provision of knowledge about addiction mechanisms. Important barriers were smokers’ fears of quitting, external environmental pressures, and inadequate counseling structures.

**Conclusion:**

The findings suggest that improving smoking cessation interventions in Germany require comprehensive strategies involving both structural adjustments in health care settings and enhanced training for tobacco treatment specialists.

**Supplementary Information:**

The online version contains supplementary material available at 10.1186/s12913-025-12646-4.

## Background

The consumption of tobacco continues to be a leading factor in preventable death and illness worldwide, posing a substantial challenge to public health [1–3]. Every year, tobacco smoking causes the premature death of about eight million people worldwide and is associated with a wide range of adverse health outcomes, including cancer, cardiovascular and respiratory diseases [2, 4]. In Germany, the prevalence of cigarette smoking remains high, with approximately 30% aged 14+ [5]. Moreover, quit rates are relatively low, with only 20% of smokers having tried to quit in the past year. In comparison, 50% of U.S. smokers made a quit attempt last year [6], while in England, this number also increased to nearly 40% [7].

Even though quitting smoking as early as possible can greatly reduce health risks, a significant proportion of people who smoke have difficulties quitting and most of the unassisted quit attempts stay unsuccessful. Even when motivated, many smokers face barriers, particularly in maintaining abstinence and preventing relapse [1, 8]. As a result, major health authorities have placed a high priority on reducing smoking rates through comprehensive tobacco control policies [9, 10]. One such policy includes the implementation of smoking cessation interventions.

In Germany, evidence-based smoking cessation treatments have shown success rates of approximately 25%. Nevertheless, among those who attempted to quit, only 13% used an evidenced-based smoking cessation method [11]. The AWMF S3 guidelines for smoking cessation in Germany recommend routine screening for tobacco use and addiction for all patients, low-threshold services such as brief phone- or app-based interventions, and psychological and pharmacological therapies for more intensive cessation support or withdrawal symptoms [12].

In a study of German oncology staff, screening patients for smoking status was common, but referrals to a smoking cessation program were rarely discussed [13]. Similar findings were observed in a representative sample of the German population, where most individuals (80.7%; 95%CI = 79–82%) who visited a physician were not given advice to quit smoking [14]. The AWMF guidelines call for the increased implementation of smoking cessation programs in health care settings, the training of health care providers, and recommend that clinical staff routinely advises smokers to quit [12]. Therefore, providing effective smoking cessation programs to help those who smoke to quit and maintain long-term abstinence is critical. To develop effective programs, we aimed to better understand the gaps and key factors (i.e., facilitators and barriers) involved in treating tobacco dependence in Germany. While quantitative studies often focus on program effectiveness, qualitative research is lacking to explore the individual experiences and social-psychological factors that impact the success or failure of smoking cessation strategies.

In this study, in-depth interviews with practitioners in the field of smoking cessation were conducted to elicit personal experiences and their views on helping people quit smoking cigarettes.

## Methods

### Sampling and recruitment

A convenience sampling method was used to select participants [15]. All participants were expected to work in smoking cessation; however, no specific training or education was required. Potential participants were identified online through the German website *Therapeuten.de*, a platform that serves as a directory for holistic therapists and consultants [16]. Inclusion criteria were current residence in Germany, fluency in German, and a minimum age of 18 years. Those who met the inclusion criteria were contacted via email. To reflect the diversity of smoking cessation experts in Germany, we selected professionals from various backgrounds, including medical doctors, social pedagogues, psychologists, addiction therapists, and social workers [17]. A sample size of 10 to 15 was chosen based on previous literature and the ability to reach saturation [18]. Participants received a compensation of €5 for their participation. The study was approved by an ethics review of the Dean of Research, Faculty of Business Administration at the University of Hamburg. Study method and results are reported following the Standards for Reporting Qualitative Research (SRQR) guidelines [19].

### Data collection

An interview guide was developed with main topic questions focusing on experiences in providing smoking cessation therapies, therapeutic strategies used by professionals, treatment factors, and service aspects to help people quit smoking. In addition, social norms and influences on smoking cessation, personal attitudes toward smoking cessation and addiction treatment were assessed. An English version can be found in the Supplements S1. The open interview structure allowed participants to express their opinions and share their experiences [20]. The use of main topic questions helped to structure the interview process, guide the research focus, and create comparable data [20, 21]. The questions were pre-tested to ensure clarity and ease of understanding. Interviews were conducted either online (i.e., via Zoom) or by telephone, were audio-recorded and later transcribed and de-identified. All participants consented to the study procedures and provided informed consent.

### Data analysis

At the beginning of the analysis, the interviews were transcribed according to Dresing and Pehl’s transcription rules [22], which involved accurately converting spoken language into written form, including detailed annotations to capture nuances such as pauses, intonation, and nonverbal cues. This ensured a comprehensive representation of the conversation. The data were then analyzed using qualitative content analysis methods. Mayring’s content analysis was applied to develop a categorization system deductively based on the main research objective of identifying facilitators and barriers in the treatment of tobacco dependence [23]. This approach involved systematic organization and interpretation of the qualitative data and helped to identify patterns, themes, and categories by coding the data, grouping the interviews into themes (e.g. categorizing), and interpreting the underlying meanings or relationships within those themes. The coding process was repeated iteratively to refine the levels of categories, which resulted in a detailed categorization system with themes and subthemes. The extracted material was paraphrased, and tables were created to enhance orientation and data presentation. This offered a clearer overview of the relationships within the themes. Based on this process, a summary heading was developed for each subcategory, and higher categories were built inductively. Once categorized, themes representing meaningful patterns in relation to the main research objective of identifying key factors for quitting smoking, were identified throughout the material. Two members of the research team independently reviewed and coded the data. The consistency between the researchers’ coding was then assessed. Any discrepancies were discussed, and if consensus could not be reached, a third member of the research team was involved until agreement was achieved. The study team consisted of health professionals with experience in prevention and health services as well as qualitative methodology, and a psychology master student.

## Results

A total of fifteen semi-structured in-depth interviews were conducted between July 2022 and November 2022. The interviews lasted between 30 and 45 min and were conducted in German.

### Sample description

Table 1 provides an overview of participants’ professions, approaches to smoking cessation, and sociodemographic characteristics, such as age, gender, and occupation. The most common professions among the participants were social educators and psychologists. Among the experts, ten were female and five were male. A variety of approaches to smoking cessation were represented: The IFT Smoke-Free program, which provides training for health professionals across Germany, is based on principles of motivational and behavioral therapy [24]. The 10-point program is an evidence-based app that doctors in Germany can prescribe for patients with tobacco addiction [25]. Both programs meet the requirements of German health insurance companies. The “Prevention For Adolescents” project was initiated by the University Medical Center Hamburg and aims to significantly reduce tobacco consumption among children and adolescents through a focus on information, counseling, treatment, as well as research and education [26].

**Table 1:** Sample characteristics, *N*=15

	**n (%)**
**Gender**	
Female	10 (67)
Male	5 (33)
**Occupation**	
Health educator	3 (20)
Psychologist	3 (20)
Dentist	1 (7)
Social scientist	2 (13)
Addiction prevention specialist	1 (7)
Psycho-oncologist	1 (7)
Alternative medicine practitioner	1 (7)
**Approach**	
IFT program^a^	7 (47)
10-point program^b^	4 (27)
Prevention For Adolescents^c^	1 (7)
Psychological counseling for addiction^d^	1 (7)
Hypnotherapy	3 (20)
**Workplace**	
Addiction counseling center	4 (27)
Own practice/ self-employed	4 (27)
Workplace addiction prevention program	3 (20)
Thoracic clinic	1 (7)
University hospital heart center	1 (7)
Lung cancer center	1 (7)
Hypnotherapy center	1 (7)
**Group Offer or Individual Offer**	
Group	9 (60)
Individual	4 (27)
Both	1 (7)

^a^The IFT program focuses on motivational and behavioral therapy

^b^The 10-point program is a digital program accessible as an app and is an evidenced-based program

^c^The Prevention for Adolescents is a smoking cessation program for young people that provides information, counseling, and treatment

^d^According to the German association for addiction counseling [27]

### Categorization system and themes

The two main categories, (1) Facilitators to smoking cessation and (2) Barriers to smoking cessation, were inductively divided into themes that addressed the characteristics of the tobacco treatment program, the individual participant, the cessation specialist, and the social environment (See Tables 2 and 3).

**Table 2:** Categorization of themes, frequency of quotes per subtheme, and sample quotes of experts’ opinions on the facilitators to help people quit smoking cigarettes

Facilitators, *N* = 89			
Theme	Subtheme	n	Sample quotes
Tobacco treatment program characteristics	Structural components (defined as costs, access, time commitment, intensity of program, group setting)	21	The social support from other…participants, the group feeling and exchange in the group setting are a supporting factor. (Participant #4) Low costs are associated with higher numbers of participants and are important for all interested smokers. (Participant #1) Telephone counseling had the advantage of not having to travel for the appointments (Participant #1)
	Content components (defined as behavioral therapies, assessment of tobacco dependence)	8	It is important to teach participants practical, relapse-preventing methods. (Participant #3) During cessation, solution strategies need to be developed for resolving potentially critical situations…moments when participants would smoke a cigarette…relapse prevention is relevant to the success of an intervention. (Participant #5)
Participant characteristics	Personal empowerment (defined as self-motivation to quit, positive outlook, experience improved physical health after quitting, resilience)	30	Self-motivation is a crucial factor, especially for treatment initiation. (Participant #1 and #2)
	Nicotine addiction knowledge (defined as understanding the multifaceted nature of nicotine addiction)	8	It is important to understand the connections between situation, addictive behavior, and neurochemical processes. (Participant #3) Noticing physical impairments and complaints can promote motivation to quit smoking. (Participant #4)
Social environment	Positive social support	7	Social and family environment is an important supporting factor. (Participant #8)
Tobacco treatment specialist characteristics	Communication skills (defined as trusting relationship and respectful interaction)	15	Conversation at eye level is relevant, it is important not to lecture smokers. (Participant #1) For the intervention leaders it is important to build a foundation of trust, as their communication skills are a crucial component of the counseling. (Participant #1) The performance of the intervention leader in motivating the participants has a significant impact on the success of the intervention. (Participant #7)

**Table 3:** Categorization of themes, frequency of quotes per subtheme, and sample quotes of experts’ opinions on the barriers to help people quit smoking cigarettes

Barriers, *N* = 57			
Theme	Subtheme	n	Sample quotes
Tobacco treatment program characteristics	Structural components (defined as high costs, the time commitment, impersonal offer)	8	High costs can be an access barrier to the intervention. (Participant #3 and #8) To my experience, over a longer period of time, only few participants attend the sessions consistently. The time commitment can complicate the success of the intervention. (Participant #1) It is complicated when patients are tackling several projects at the same time, of which smoking cessation is only one. (Participant #4) Phone offered programs can be too impersonal (Participant #10)
Participant characteristics	Cognitive barriers to change (defined as a fear of failure, fear of loss, fear of negative emotional consequences, dysfunctional attitudes and beliefs, lack of information)	27	There is the fear of experiencing a permanent lack. (Participant #5) Participants can fear remaining unsuccessful after several attempts (Participant #8) Emotional factor can lead to not even trying to quit smoking (Participant #1) Participants believe that nicotine satisfies various needs (Participant #7) Participants frequently compare smoking to alcohol in road traffic…in this comparison the risk of tobacco use seems secondary (Participant #2)
	Comorbid condition	7	There are difficulties in smoking cessation…with depression…nicotine also has an antidepressant effect and can work as a psychological anchor. (Participant #1) Additional diagnoses in the psychiatric area complicate the success of the intervention…if an additional diagnosis is present, I first advise for the treatment of this diagnosis, then proceed to smoking cessation. (Participant #3)
Social environment	External pressure, including frequent exposure to people who smoke	15	External convincing to quit smoking has a low probability of success. (Participant #1) Participants’ mindset needs to change from ‘I shouldn’t smoke’ to ‘I don’t need to smoke’, thus leaving behind perceived external pressure. External advice is problematic. (Participant #6)

#### Main category 1 - Facilitators (f) to smoking cessation

*fA. Tobacco treatment program characteristics* discussed were categorized into *structural* (fA.1.) and *content* (fA.2.) components.

##### fA.1.

A facilitating *structural component* of a tobacco treatment program included costs. Experts mentioned the value of offering tobacco treatment programs free of charge to reduce barriers to participation and increase accessibility. From their experience, if costs were particularly low, more people would consider participating, and higher participation rates could be observed. In contrast, it was mentioned that “…bearing high costs can increase motivation and ultimately promote the success of the intervention“ (Participant #5). It was believed that a high fee could give the perception of more value to a program. Other structural key components were the time commitment and intensity of a program. Flexible scheduling, with dates and times chosen by the participants, was considered important by the experts. This flexibility would allow individuals to fit appointments into their personal schedules, making it easier for them to attend regularly and stay engaged. Flexible scheduling could also increase their sense of autonomy and commitment to the program. Additionally, five experts stressed the importance of intensive, structured and closely monitored interventions. These included regular check-ins with a consultant, close coordination with experts, and structured group meetings at set intervals to maintain motivation and prevent relapse. Experts also advocated for interventions that began with an initial motivational counseling session, immediately followed by courses to ensure a seamless transition and keep participants engaged. A group setting was found to be another important structural component. Five experts endorsed smoking cessation in a group setting, citing the benefits of social support and interaction among participants. The role of negative examples in motivating the group was also discussed. Seeing group members struggle to quit could highlight the challenges of smoking cessation for others and encourage them to avoid similar pitfalls while staying committed to their own goals.

##### fA.2

In terms of *content components*, the experts highlighted the value of behavioral therapy, which incorporates techniques of behavioral change and solution-based strategies that can be integrated into daily life. For instance, the use of relaxation, concentration, and breathing techniques was mentioned as a way to improve the success of quitting smoking. To prevent relapse, the experts emphasized the importance of developing strategies to manage critical situations after quitting—such as moments when participants would typically smoke a cigarette. Another important content component was the assessment of tobacco use and tobacco dependence. One expert pointed out the general lack of comprehensive assessment and “good diagnostics” (Participant #1) in addiction treatment, advocating for improved addiction counseling by other health professionals, such as general practitioners.

*fB. Participant characteristics* were further categorized into two subthemes: (1) *personal empowerment* (fB.1.), and (2) *nicotine addiction knowledge* (fB.2.).

##### fB.1

*Personal empowerment* included having the self-motivation to quit, which was most frequently mentioned by experts. Experts believed that entering a smoking cessation program requires participants to exhibit some level of self-motivation, involving intrinsic motivation and initiative, which were seen as crucial throughout the cessation process. Participant motivation was cited as a key factor for initiating treatment, maintaining participation, and ultimately achieving success in smoking cessation interventions. Experts stressed the importance of accurately assessing participants’ motivation at the start of the intervention and monitoring it throughout the process. Two experts considered motivation a critical factor in determining whether individuals who smoke should participate in a tobacco dependence treatment program. The experts’ work experience and feedback from participants reinforced the idea that successful quit attempts were primarily made by those who were motivated to quit. Intervention leaders played a key role in supporting participants by helping them to recognize their personal benefits of quitting and emphasizing that participation in the intervention was entirely voluntary. A positive outlook on the future was also discussed by experts, focusing on the importance of envisioning a non-smoking future self and developing positive internal images, such as a “relaxed non-smoker” (Participant #1), or other personal ideals. According to the experts, these positive internal images can have a “magnetic effect” (Participant #12) and serve as a driving force for participants. Additionally, the experts emphasized the positive impact of experiencing improved physical health after quitting smoking. Experts believed that the perception of physical impairment, illnesses, and complaints could increase motivation to quit smoking by stimulating the desire for a better physical health. Experts also highlighted the role of resilience, stating that 'tolerance of frustration can facilitate success' (Participant #13) in smoking cessation.

##### fB.2

The experts emphasized the importance of *nicotine addiction knowledge* which includes understanding the mechanisms of action of addictive substances and the links between situations, addictive behavior and neurochemical processes for quitting success and individual triggers for successful smoking cessation. It was recommended that developing an addiction model together with participants may help them recognize their personal behavioral patterns. Experts observed that once participants fully grasped their addiction to nicotine, they were able to reclaim self-efficacy and autonomy after quitting.

*fC. Social environment*, particularly the supportive role of social networks and the family environment, was seen as having a positive impact on smoking cessation. For example, a partner’s perspective on smoking and cessation methods was considered influential within relationships. A stable social network was seen as generally advantageous, as it reduced the need for smoking as a social activity, reducing the pressure to smoke in social settings and making it easier to quit.

*fD. Characteristics of the tobacco treatment specialist* was regarded as crucial in providing effective guidance during the process. *Communication skills* that fostered trust and respectful interactions were frequently mentioned during the interviews. Experts highlighted the importance of communication skills, particularly the use of empathy to build trust, motivational statements, and effective counseling strategies. Good communication was perceived as essential for promoting participants’ intrinsic motivation and reinforcing any existing internal ambivalence about smoking. In fact, the intervention leader was sometimes perceived as the key influence, even referred to as a “motivational clown” (Participant #7) for their ability to inspire participants. The benefits of respectful interaction and the importance of avoiding a dictatorial approach were also emphasized. Experts emphasized that conversation should take place “at eye level” (Participant #1), especially when offering advice.

#### Main category 2 - Barriers (b) to smoking cessation

*bA. Tobacco treatment program characteristics*, such as the high costs was a *structural component* discussed in several expert interviews. Similarly, the time commitment required for smoking cessation programs was identified as another obstacle to successfully quitting. Experts observed that many participants dropped out of smoking cessation sessions over time due to challenges in attending regularly. It was perceived as a barrier to quitting if those in a smoking cessation program also had other goals, such as a challenging career, other health or personal life goals, and smoking cessation was only one project among others and thus lacked priority. The *content component* of a lack of personalization, such as programs not tailored to the individual needs of the person who smokes, was identified as a barrier to successful smoking cessation. For example, telephone interventions were mentioned as impersonal, due to the absence of face-to-face interaction or the challenges of expressing emotions when non-verbal cues are limited.

*bB. Participant characteristics* that were viewed as barriers were labeled *cognitive barriers to change* (bB.1.) and *comorbid conditions* (bB.2.).

##### bB.1

Fears, as a *cognitive barrier to change*, was a common theme in the interviews as significant barriers to quitting. A fear of failure before starting a tobacco dependence treatment program could prevent an attempt to quit altogether and was viewed as a major barrier to participation in a tobacco treatment program. For many experts, this fear was believed to have stemmed from previous failed attempts, with questions such as “How will I manage?” (Participant #6) reflecting their underlying concerns. Another issue raised by experts was the fear of loss and the strong psychological dependency on tobacco smoking. People who smoked were often afraid of losing something important in their lives and of “experiencing a permanent lack” (Participant #5), which discouraged them from attempting to quit. According to experts, the stronger the psychological attachment to smoking, the higher the risk of relapse. For some, the cigarette was perceived as a “good friend” (Participant #15) that provided “comfort and coziness” (Participant #11). Experts also discussed the fear of negative emotional consequences, such as withdrawal symptoms, which could be “overwhelming” (Participant #8), and the fears of increased stress, irritability, and reduced cognitive ability. In addition, experts found that participants’ dysfunctional attitudes and beliefs interfered with their ability to quit smoking. One such belief was that nicotine satisfies a variety of needs. Shifting patients’ perceptions of nicotine’s role in fulfilling these needs and raising awareness of addiction mechanisms posed significant challenges in tobacco dependence treatment. A pleasure-oriented attitude toward life also made quitting more difficult. Experts reflected on how many participants think ‘I only live once, so I smoke’ (Participant #2) and ‘why should I stop now?’ (Participant #7) which served as justification for continued consumption. Another barrier to smoking cessation discussed by experts was the lack of information about the health risks associated with smoking among their clients. In their professional experience, closing this knowledge gap required effective, evidence-based interventions to support smoking cessation.

##### **bB.2**

The expert interviews highlighted the presence of *co-morbid conditions*, where mental illness alongside addiction was seen as a significant barrier to smoking cessation. Experts suggested that treating the mental illness should take priority, as addressing a psychiatric disorder could complicate the treatment of tobacco dependence if both were managed simultaneously.

*bC. Social environment* was a barrier, as well as a facilitator. Experts talked about the difficulty of participants taking part in tobacco treatment programs due to *external pressure* from society, rather than from their own intrinsic motivation. Experts believed that providing participants with information about available tobacco treatment options was sufficient to encourage participation, rather than pressure someone to quit. Lastly, several experts discussed exposure to secondhand smoke from family members and being in social settings where smoking is common as significant barriers to quitting.

## Discussion

During the interviews, the experts identified several factors that may contribute to the quitting process. They considered facilitators and barriers to successful smoking cessation in several areas and under different aspects, such as the structure and content of the tobacco treatment program itself, their own role as smoking cessation specialist, and the characteristics of the participants including their cognition, living conditions, and social environment.

The characteristics of a tobacco treatment program can either support or hinder successful smoking cessation. Experts highlighted the advantages of flexible scheduling, noting that the effectiveness of a program often depends on its compatibility with participants’ personal commitments and work schedules. Similarly, previous research has identified the lack of flexibility in smoking cessation program as a barrier to quitting [28]. However, experts also stressed the importance of maintaining a high level of interaction between the participant and specialist, emphasizing that engagement in the smoking cessation program should be a priority for participants. This aligns with existing research showing that face-to-face or telephone counseling is more effective than written support alone [1, 29].

Based on experts’ experiences, more frequent and closer appointments lead to higher success rates in quitting. Integrating digital tools into a cessation program can help maintain a high level of program intensity while reducing the time commitment required. AI-based interventions, though still emerging, show great potential for enhancing engagement and adherence [30].

The integration of digital tools into conventional counseling and pharmacological treatment has been shown to double abstinence rates [1], making them a valuable addition to smoking cessation programs. Increasing the accessibility of smoking cessation programs to participants is important to experts. With new digital technologies, including AI-driven programs, services to help people quit are now more readily available and can help to offer effective support and education, positively influencing self-efficacy and motivation to quit [30, 31]. Due to these various benefits, they should be incorporated into healthcare guidelines.

Research indicates that the mode of delivery does not impact long-term adherence to smoking cessation [32], even making digital interventions a suitable and accessible alternative for individuals with reduced mobility or those must travel long distances [12]. However, some participants require additional support, and the importance of worksite-based cessation programs has been highlighted in other studies [32].

A lack of personalization, such as interventions that are too broad or too general, was mentioned as a barrier to quitting. Similarly, research has shown that seminars with impersonal content decrease smokers’ motivation to quit [28]. Consistent with this, other studies have found that delivering personal support– whether face-to-face or by telephone [29]– and providing individualized treatment plans [33] offer important benefit for smoking cessation.

Theoretical frameworks such as the Transtheoretical Model suggests that tailored interventions based on a person’s stage of change—pre-contemplation, contemplation, or preparation—can help overcome motivational barriers [34, 35]. By assessing a smoker’s readiness to quit, counselors can provide personalized feedback and targeted support, making cessation efforts more effective. This stage-based approach ensures that individuals receive guidance that “meets them where they are”, helping them progress toward quitting.

Having a tobacco specialist available at all times was seen as helpful [28], but this approach may be impractical and too time-intensive for a single specialist managing multiple clients. New artificial intelligence tools are being tested to provide a “virtual counselor on-demand”, but whether personalization can be achieved is not yet certain [30, 36]. The relationship between the participant and the treatment specialist is viewed as highly important, and as voiced by many of the experts in this study, an intervention should be tailored to the individual needs and circumstances of the participant.

Positive social support, whether provided through a tobacco cessation program in a group setting or from family and friends, was also identified by experts as a crucial factor in helping people quit smoking. Research has shown that participants benefit from peer support and activities that encourage interaction [37]. In a previous study, participants in smoking cessation programs noted the advantage of having a peer to quit with, as they could offer mutual support throughout the process [38]. People who smoke find it helpful to learn from one another and share their ideas and strategies for successful cessation with other smokers who are trying to quit [1, 8, 39]. Identifying negative social support or triggers for smoking is also crucial in the quitting process. Other research has highlighted that a smoking-friendly environment can be a significant barrier to smoking cessation [31, 40]. In settings where smoking is a social norm, individuals who wish to quit may feel pressure to conform to the group [31, 41]. By recognizing these triggers, individuals can develop coping mechanisms or avoidance strategies to support their efforts to quit.

Furthermore, addressing triggers are one-way participants can increase their personal empowerment, or self-efficacy, to quit smoking. Experts emphasized the importance of having cognitive skills, such as the motivation to quit, a positive outlook, focusing on positive experiences after quitting, and resilience– skills that are particularly valuable when facing urges or relapses, which are common during the quitting process. Experts also highlighted the importance of behavioral therapies, such as using alternative relaxation strategies like breathing or concentration exercises, to manage symptoms such as cravings and tension. Self-efficacy, the belief in one’s own abilities [42], is a widely recognized factor in behavioral therapies and is considered highly important in promoting the motivation to quit. In examining correlates of self-efficacy in a community sample of people who smoked, one study found perceived control over withdrawal symptoms and the ability to manage cravings stimulated by positive affect or social smoking cues correlated with self-efficacy [43]. Tobacco treatment programs should continue to increase self-efficacy by focusing on helping participants deal with social situations, triggers, and withdrawal symptoms.

Notably, any mention of nicotine replacement therapies (NRT) or medications (such as bupropion) to aid smoking cessation was missing during the interviews. The “gold standard” for treating tobacco dependence typically includes pharmacotherapy combined with behavioral support [44], yet this combination of treatments was not part of the conversations with the experts. Further research is warranted to understand why experts were not inclined to bring up the use of pharmacotherapy to quit smoking in their participants and if participants would be interested in using NRT or medication for stopping smoking.

Our sample did not include psychiatrists or physicians, despite their important role in tobacco cessation and patient counseling. This was due to our convenience-based sampling method, as no physicians responded to our call for participation. Additionally, it was not assessed whether the experts followed evidence-based cessation approaches or held specific qualifications. For instance, three participants reported using hypnotherapy, a method deemed ineffective in Cochrane reviews [28, 45, 46]. It is possible that practitioners using hypnotherapy are less likely to adhere to evidence-based treatments, including pharmacological support [47, 48]. However, as to date hypnotherapy remains a practice used in the field, we chose to include these participants [46].

One recommendation that experts felt was missing was the systematic assessment of tobacco dependence by other health professionals. The AWMF guidelines recommend routine screenings for tobacco use and addiction, for example by using the Fagerström test [12, 49], along with brief interventions like the 5 A’s approach [44] in clinical settings, which have been proven to be an effective and cost-efficient approach for all patients [12]. Although the interviewed experts viewed co-morbid conditions a barrier to smoking cessation, addressing smoking habits can improve overall health outcomes. In cases of somatic diseases related to tobacco use, the AWMF guidelines advise that smoking cessation should be promoted in acute care settings [12].

Additionally, patients with psychiatric disorders should receive the same cessation advice as other smokers, including psychosocial, psychotherapeutic, and medical approaches. For patients with current or past episodes of depression, cessation interventions should include addressing depression-related components, such as coping with negative moods. In the context of schizophrenia, smoking cessation efforts should focus on patients who are in a stable disease status [12]. Likewise, scientist from other countries such as Ireland, Australia or Spain recommend treating both tobacco addiction and psychiatric comorbidities simultaneously [50].

Our findings highlight the need for improved accessibility to smoking cessation programs, as experts emphasized the importance of frequent and intensive support. Hospital-level policies could integrate smoking cessation interventions as a routine part of patient care, ensuring that every smoker receives brief advice and referral options. Additionally, telehealth services could enhance accessibility, particularly for individuals in rural areas or those with limited mobility.

Making smoking cessation therapies more affordable through health insurance coverage or subsidized programs would further reduce barriers to access. Ultimately, increased funding is necessary to support evidence-based cessation services and expand their reach.

### Strengths and limitations

When discussing the results of this study, several limitations should be considered. The findings are based on the individual views of experts, which are shaped by their professional experiences. Further research is needed to validate these insights and assess their broader effectiveness. To reduce potential bias in interpretation, a methodological approach was used, with two researchers conducting the interviews and performing ongoing checks throughout the analysis, ensuring quality control. Other than the aim of identifying facilitators and barriers to smoking cessation, there was no pre-existing theoretical framework guiding the thematic analysis [51]. The data were organized solely based on the information gathered from the interviews. The use of a convenience sample may limit the generalizability of the results, and the possibility of selection bias should also be considered. However, the experts represented a range of approaches to smoking cessation and professions reflective of those providing smoking cessation programs in Germany.

Because the interviews were mainly concentrating on professional experiences, they did not include questions on additional demographic data of the participants, their own smoking status, or personal experience with smoking cessation (e.g., their own attempts or those of family members). Such factors and experiences could shape their perspectives on smoking cessation and should be considered in future research.

While the concept of data saturation [18] carries the risk of missing significant additional information, we are confident that the most important themes were effectively captured. This was achieved by developing comprehensive topic guides in advance and allowing sufficient time for participants to reflect and respond. The interviews were conducted exclusively with German health care providers, which may limit the generalizability of the findings to other countries and health care systems. However, key aspects such as the importance of low-cost cessation programs, personalized interventions, and addressing fears and withdrawal symptoms are widely relevant. Therefore, the findings may offer valuable insights that can be transferable to different contexts, other countries and systems.

An important strength of this work is the inclusion of experts from diverse clinical backgrounds, providing a multidisciplinary perspective on smoking cessation. To date, there has been conducted limited research on the quality of smoking cessation interventions. To the best of our knowledge, this is the first study to assess expert views on smoking cessation and to collect work experiences of professionals in Germany. Qualitative research has the great advantage of being able to collect reflective thoughts and explore people’s personal opinions on a topic. This approach provides a deeper insight into complex aspects of experience [52]. In conclusion, this qualitative study provides valuable insights and suggestions for improving smoking cessation interventions.

## Conclusion

Cigarette smoking remains a major concern, especially in high-income countries such as Germany [53]. This study provides a deeper insight into the key factors of quitting smoking. In-depth expert interviews with professionals working in the field of tobacco dependence and smoking cessation helped to identify facilitators and barriers to achieving smoking cessation.

The results highlighted the importance of the structure (i.e., costs and intensity of a program) and components (i.e., behavioral therapies) of a smoking cessation program, participants’ personal empowerment or self-efficacy to quit, the communication skills of the tobacco treatment specialist, and a supportive social environment. Tobacco treatment programs should be low cost and accessible, tailored to individual needs, and address fears, beliefs, and how to handle triggers and withdrawal symptoms. Lastly, smoking status should be addressed in all patient populations. Further studies should determine the consistency of expert opinions with the opinions and personal experiences of people who smoke cigarettes.

## Supplementary Information

Supplementary Material 1. S1 Interview guide for smoking cessation experts– English version

## Data Availability

All relevant data are included in the analysis. The datasets analyzed during the current study are available from the corresponding author on reasonable request.
