# Supplementary material for: Facilitators and barriers to smoking cessation: a qualitative study among health professionals in Germany
Source: BMC Health Serv Res. 2025 Apr 1;25:483. doi: 10.1186/s12913-025-12646-4 (PMC11959832; doi:10.1186/s12913-025-12646-4)
Supplement: Supplementary file 1 — Supplementary Material 1. S1 Interview guide for smoking cessation experts– English version [file 12913_2025_12646_MOESM1_ESM.docx]

**Supplementary File 1**

**Interview guide for smoking cessation experts**

**General experience**

Main question: Do you have any experience in offering smoking cessation aid? If yes, explain.

Probing questions:

What criteria do you use to determine who you would recommend/refer/offer for smoking cessation treatment?

What type of therapy do you recommend? Why? And for how long?

How often do you talk to people who smoke about quitting (e.g., in a given month on average)?

How often do you follow-up with people regarding their smoking habits?

Of those who you talk to about quitting, how many or what percentage receive smoking cessation therapy (either counseling, a class, enroll in a program, take NRT or medication)?

**Treatment characteristics**

Main question: In thinking about helping people to quit smoking, what are important treatment factors? (e.g., costs, group therapy, in-person therapy, medication, duration of therapy, side effects of medication, drug interactions, peer and/or family support, effectiveness) Explain.

Probing question: How would you describe a patient who is "dependent on smoking"? How do you asses this?

**Costs**

Main question: How much should smoking cessation therapies be and why?

Probing question: What should be covered? and for how long? and to whom (i.e., all smokers, severely dependent smokers)?

**Perceived access and barriers to smoking cessation therapies**

How do people access smoking cessation therapies you are offering (e.g., online, thru Dr. referral, from work)?

**Emotions**

Are there any emotions your clients report when thinking about quitting smoking? Explain.

**Non-approved cessation methods: E-cigarettes and heated tobacco products**

Main question: What are your thoughts on people reporting using e-cigarettes or other types of tobacco products to help them quit smoking?

Probing question: Information about e-cigarettes: People have reported using non-approved devices such as e-cigarettes to help them quit smoking cigarettes. What do you think about this?
